# Keratin 17 Suppresses Cell Proliferation and Epithelial-Mesenchymal Transition in Pancreatic Cancer

**DOI:** 10.3389/fmed.2020.572494

**Published:** 2020-11-26

**Authors:** Yong Zeng, Min Zou, Yan Liu, Keting Que, Yunbing Wang, Changan Liu, Jianpin Gong, Yu You

**Affiliations:** ^1^Department of Emergency, The Second Affiliated Hospital of Chongqing Medical University, Chongqing, China; ^2^Department of Gastroenterology, West China Hospital of Sichuan University, Sichuan, China; ^3^Department of Gastroenterology, The Fifth People's Hospital of Chengdu, Chengdu, China; ^4^Department of Hepatobiliary Surgery, The Second Affiliated Hospital of Chongqing Medical University, Chongqing, China

**Keywords:** K17, epithelial-mesenchymal transition, pancreatic cancer, keratins, carcinogenesis

## Abstract

Keratin 17 (K17), a member of type I acidic epithelial keratin family, has been reported to be upregulated in many malignant tumors and to be involved in promoting the development of tumors. However, the precise role of K17 in progression of pancreatic cancer is still unknown. In this study, we found that K17 expression was highly expressed in pancreatic cancer tissues and cell lines and that upregulated expression was associated with the pathological grade and poor prognosis. K17 expression served as an independent predictor of pancreatic cancer survival. Meanwhile, we showed that knocking down K17 induced pancreatic cancer cell proliferation, colony formation and tumor growth in xenografts in mice. However, K17 upregulation inhibited pancreatic cancer cell proliferation and colony formation. Further mechanistic study revealed that K17 knockdown promoted cell cycle progression by upregulating CyclinD1 expression and repressed cell apoptosis. However, K17 upregulation suppressed cell cycle progression by decreasing CyclinD1 expression, and induced apoptosis by increasing the levels of cleaved Caspase3. In addition, K17 knockdown promoted pancreatic cancer cell migration and invasion, but K17 upregulation suppressed cell migration and invasion. Moreover, knocking down K17 promoted epithelial-mesenchymal transition (EMT) in pancreatic cancer cell by inhibiting E-cadherin expression and inducing Vimentin expression, and the effects of K17 upregulation were opposite to that of K17downregulation. Taken together, our findings suggest that K17 functions as a potential tumor suppressor, even though it is upregulated in pancreatic cancer.

## Introduction

Pancreatic cancer is a highly lethal malignancy with a median survival of <6 months, and it is the fourth leading cause of cancer-related death in Western societies (1–3). Although diagnosis and treatments for most cancers have witnessed immense progress over recent decades, only 9.7% of pancreatic cancer patients are diagnosed at an early stage, and the 5-year survival rate of patients with pancreatic cancer is only 8% (3). Therefore, it is of great importance to clarify the molecular pathogenesis of pancreatic cancer to develop novel strategies for the diagnosis and treatment of this malignancy.

Keratins, a family of intermediate filament proteins, are essential for maintaining the structural stability and integrity of keratinocytes and protecting epithelial cells from damage (4). Currently, 54 functional keratins have been identified and are classified as type I or type II based on their pH (5). Keratin 17 (K17), a type I Keratin, is mainly expressed in stem cells of the skin appendages, embryonic ectodermand endocervical mucus or reserve cells in the epithelium (6). Under physiological and pathological conditions, K17 expression can be rapidly induced. For instance, the expression levels of K17 are increased during the early stage of human skin injury, and its high expression is of great benefit to wound healing (7). Recent evidence suggests that K17 is upregulated in various types of tumors, including oral cancer, non-small cell lung cancer, basal cell carcinoma and cervical cancer, and when overexpressed, K17 acts as an oncogene (8–11). In addition, previous studies have demonstrated that K17 mRNA is highly expressed in pancreatic ductal adenocarcinoma (PDAC) (12, 13). Roa-Peña et al. (14) demonstrated that increased K17 expression is associated with shorter survival of patients with PDAC. However, the role of K17 in pancreatic cancer has not yet been reported.

Here, we investigated the precise role of K17 in pancreatic cancer progression. We found that K17 was upregulated in pancreatic cancer tissues compared to adjacent nontumor tissues, and high expression of K17 was associated with relatively poor survival in pancreatic cancer. Interestingly, we revealed that K17 repressed pancreatic cancer cell proliferation by inducing cell cycle arrest and apoptosis, and inhibited pancreatic cancer cell migration and invasion by suppressing epithelial-mesenchymal transition (EMT). This is the first report of K17 as a tumor suppressor gene, although the precise mechanism of its action requires further study.

## Materials and Methods

### Patients and Tissue Samples

Tumor tissues were collected from 94 pancreatic cancer patients who underwent curative surgery from September 2004 to December 2008 at the Second Affiliated Hospital of Chongqing Medical University. This study was approved by the Research Ethics Committee of the Second Affiliated Hospital of Chongqing Medical University (China). In addition, informed consent was obtained from all patients prior to surgery. All patients who had previously received chemotherapy or radiotherapy were excluded. Clinical and pathological information was collected and is listed in Table 1. The overall survival of patients data was acquired at 2-month intervals through telephone calls. The follow-up data stopped being collected in December 2011.

**Table 1:** Correlations between clinicopathological characteristics and K17 expression in pancreatic cancer patients.

**Variable**	**All cases**	**K17**		***P-*value**
		**Low expression**	**High expression**	
Age				1.000
≤ 60	47	35	12	
>60	47	35	12	
Gender				0.809
Male	57	42	15	
Female	37	28	9	
Tumor size (cm)				0.562
≤ 4	58	42	16	
>4	36	28	8	
Pathological grade				0.005^**^
I + II	71	58	13	
III + IV	23	12	11	
TNM stage				0.337
I–IIA	51	40	11	
IIB–IV	43	30	13	
Tumor stage				0.606
T1 + T2	74	56	18	
T3 + T4	20	14	6	
Lymph node metastasis				0.465
Negative	53	41	12	
Positive	41	29	12	
Distant metastasis				0.422
M0	92	69	23	
M1	2	1	1	

** *Statistically significant (P < 0.01)*.

### Immunohistochemistry (IHC)

IHC analysis was performed to detect the expression of K17 and Ki67 protein in tissues as previously reported (15). Paraffin-embedded tissues were cut into 4-μm-thick sections. The sections were then baked at 70°C for 2 h, dewaxed in xylene, hydrated in graded alcohol, and placed in 3% hydrogen peroxide for 20 min to block endogenous peroxidase activity. Next, tissue slides were subjected to microwave treatment for antigen retrieval and then were incubated with 10% normal goat serum for 20 min. Subsequently, tissue slides were incubated with a rabbit monoclonal K17 antibody (ab109725, Abcam, Cambridge, MA, UK) or a rabbit polyclonal Ki67 antibody (27309-1-AP, ProteinTech Group, Inc., Wuhan, China) overnight at 4°C. Tissue slides were then incubated with a biotin-conjugated secondary antibody (SA00004-2, ProteinTech Group, Inc., Wuhan, China) for 1 hat room temperature. Finally, tissue slides were stained with 3,3′-diaminobenzidine (DAB) (K5007, Dako Corporation, Glostrup, Denmark). IHC scores were computed as described previously (16). Staining intensity was graded as follows: negative = 0, weak-positive = 1, moderate-positive = 2 or strong-positive = 3. The positive rate score was graded as follows: zero represented positive areas of 0–5%, one represented positive areas of 6–25%, two represented positive areas of 26–50%, three represented positive areas of 51–75% or four represented positive areas of 76–100%. The final score was calculated by multiplying the staining intensity and the positive rate scores.

### Cell Culture

The normal pancreatic ductal epithelial cell line HPDE6-C7 and the human pancreatic cancer cell lines CFPAC-1, MIA PaCa-2, PANC-1 and SW990 were obtained from the Cell Bank of the Chinese Academy of Sciences (Shanghai, China). HPDE6-C7, MIA PaCa-2 and PANC-1 cell lines were cultured in DMEM (Gibco, Grand Island, NY, USA) containing 10% fetal bovine serum (FBS) (Gibco, Grand Island, NY, USA). CFPAC-1 cells were cultured in IMDM (Gibco, Grand Island, NY, USA) supplemented with 10% FBS. SW1990 cells were maintained in IMDM (Gibco, Grand Island, NY, USA) containing 10% FBS.

### Lentivirus Construction and Infection

A K17 shRNA (5′-CCGGCACCTGACTCAGTACAAGAAACTCGAGTTTCTTGTACTGAG TCAGGTGTTTTTTG-3′) and a negative control (5′-CCGGTTCTCCGAACGTGTCACGTTT CAAGAGAACGTGACACGTTCGGAGAATTTTTG-3′) were designed and synthesized by GenePhama (Shanghai, China). Each shRNA was then subcloned into a pLV-U6-shRNA-CMV-EGFP-2A-Puro lentivirus vector and packaged using 293T cells by Sesh-biotech (Shanghai, China). Finally, these lentiviruses were concentrated by ultracentrifugation at 82,700 × *g* for 2 h, and the virus with the K17 shRNA was referred to as LV-K17 RNAi. The full-length cDNA of human K17 (NM_000422.2, 1299 bp) was synthesized and subcloned into a pLV-CMV-gene-PGK-EGFP-T2A-Puro lentivirus overexpression vector by Sesh-biotech (Shanghai, China). Then, the lentivirus overexpression vector was packaged using 293T cells by Sesh-biotech (Shanghai, China). Subsequently, these lentiviruses were concentrated by ultracentrifugation at 82,700 × *g* for 2 h, and the final product was referred to as LV-K17 ov.

To establish stable K17 konckdown cell lines, SW1990 and CFPAC-1 cells were cultured in 6-well plates. When SW1990 and CFPAC-1 cells were 40% confluent, they were infected with LV-K17 RNAi at an MOI (multiplicity of infection) of 20 in the presence of 8 μg/ml polybrene, and then they were selected by treatment with puromycin (2 μg/ml and 5 μg/ml) for 3 weeks. To establish stable K17 overexpressing cell lines, HPDE6-C7 and PANC-1 cells were cultured in 6-well plates. When PANC-1 cells were 40% confluent, they were infected with LV-K17 ov at an MOI of 30 in the presence of 8 μg/ml polybrene, and then they were selected by treatment with puromycin (2 μg/ml and 3 μg/ml) for 3 weeks.

### Western Blot Analysis

Cells were washed with PBS and lysed by RIPA lysis buffer (Beyotime, Shanghai, China) with 1 mM phenylmethyl sulfonylfluoride (PMSF) (Beyotime, Shanghai, China). Protein concentrations were then quantified using a BCA protein assay kit (Beyotime, Shanghai, China). Subsequently, the proteins were denatured at 100°C for 10 min, loaded and separated on 10% SDS-PAGE gels and blotted onto PVDF membranes. Tris-buffered saline that contained 5% nonfat powdered milk was used to block non-specific binding for 1 h at room temperature. The membranes were incubated at 4°C overnight with the following antibodies: rabbit monoclonal K17 (ab109725, Abcam, Cambridge, MA, UK), rabbit polyclonal cleaved Caspase3 (ab2302, Abcam, Cambridge, MA, UK), rabbit polyclonal CyclinD1 (26939-1-AP, ProteinTech Group, Inc., Wuhan, China) and rabbit polyclonal GAPDH (10494-1-AP, ProteinTech Group, Inc., Wuhan, China). After washing, the membranes were incubated with HRP-conjugated secondary antibodies for 2 h at room temperature. Finally, ECL reagents (Thermo Scientific, Rockford, IL, USA) were used to detect the protein signals.

### Quantitative Real-Time PCR (qRT-PCR)

Total RNA was extracted from cells using TRIzol reagent (Invitrogen, Carlsbad, CA, USA) according to manufacturer's instructions. UV absorbance spectroscopy was used to determine the RNA purity and quantity. The RNA was then reverse-transcribed to generate cDNA using M-MLV Reverse Transcriptase (TaKaRa, Dalian, China). A SYBR Premix Ex Taq kit (TaKaRa, China) was used to analyze the gene expression levels. Primers for K17 were 5′-CGTGACCAGTATGAGAAG-3′ (forward) and 5′-TTCAGTTCCTCTGTCTTG-3′ (reverse). Primers for E-cadherin were 5′-CGGACGATGATGTGAACACC-3′ (forward) and 5′-TTGCTGTTGTGCTTAACCCC-3′ (reverse). Primers for Vimentin were 5′-GAGTCCACTGAGTACCGGAG-3′ (forward) and 5′-ACGAGCCATTTCCTCCTTCA-3′ (reverse). Primers for GAPDH were 5′-TGACTTCAACAGCGACACCCA-3′ (forward) and 5′-CACCCTGTTGCTGTAGCCAAA-3′ (reverse). GAPDH was used as an internal control. The relative expression of genes was calculated using the 2^−ΔΔCt^ method (17).

### Cell Proliferation Assay

A Cell Counting Kit-8 (CCK-8) (Beyotime, Shanghai, China) assay was used to assess cell proliferation. SW1990, CFPAC-1, HPDE6-C7 and PANC-1 cells were seeded in 96-well plates (2,000 cells/well) and then were incubated for 0, 1, 2, 3, and 4 d. Following incubation, 10 μl of CCK-8 solution was added to each well. After incubation for 3.5 h, the number of viable cells was measured by a microplate reader at 450 nm.

### Colony Formation Assay

To analyze cell growth, colony formation assays were performed. SW1990, CFPAC-1, HPDE6-C7 and PANC-1 cells were seeded in 6-well plates at a density of 500 cells/well and then were incubated for 14 d. Subsequently, 0.05%crystal violet was used to stainfor 20 min. Cell colonies were counted using a light microscopy.

### Cell Cycle Assay

The cell cycle distribution was measured by propidium iodide (PI) staining. The cells were harvested and washed with cold PBS. Next, the cells were fixed by incubation with 70% ice cold ethanol at 4°C overnight. After washing in cold PBS, the cells were resuspended in 100 μg/ml RNase and then were stained with 50 μg/ ml PI (Sigma-Aldrich, St. Louis, MO, USA) for 30 min at 4°Cin the dark. Finally, the cells were analyzed using a flow cytometry (Becton Dickinson, Franklin Lakes, NJ, USA).

### Apoptosis Assay

For apoptosis assays, SW1990, CFPAC-1, HPDE6-C7 and PANC-1 cells were harvested, centrifuged at 400 × *g* for 5 min, and washed with cold PBS. Subsequently, the cells were stained with an Annexin V/PI detection kit (BD Bioscience, San Diego, CA, USA) following the manufacturer's protocol. Finally, the stained cells were analyzed using flow cytometry (Becton Dickinson, Franklin Lakes, NJ, USA).

### Wound Healing Assay

For cell migration assays, SW1990, CFPAC-1, HPDE6-C7 and PANC-1 cells were seeded in 12-well plates at a density of 2 × 10^5^ cells/well and then were incubated for 24 h. When the cells reached 90% confluence, sterile pipette tips were used to scratch a wound on the surface of the confluent cell monolayer. The speed of wound closure was monitored after 48 h by measuring the distance of the wound from 0 h.

### Cell Invasion Assay

For cell invasion assay, Matrigel-coated Transwell cell culture chambers (BD Biosciences, Bedford, MA, USA) with membrane pore size of 8.0 μm (Corning, Tewksbury, MA, USA) were used. A total of 5 × 10^4^ cells in 100 μl of serum-free medium were added to each insert, and 500 μl of medium with 10% FBS was added to the lower chambers. After 48 h of incubation, cells on the upper side of the filter were removed, and those that invaded through the filter into the lower side of the membrane were fixed and stained with crystal violet. The number of cells that invaded was calculated under a microscope.

### Immunofluorescence

SW1990, CFPAC-1, HPDE6-C7 and PANC-1 cells were isolated and plated on round coverslips. After 24 h of incubation, cells were washed with PBS and fixed in 4% paraformaldehyde for 15 min. After washing, cells were permeabilized with 0.2% Triton X-100 for 10 min. Subsequently, cells were blocked with 5% normal goat serum for 30 min. Next, a rabbit polyclonal E-cadherin antibody (20874-1-AP, ProteinTech Group, Inc., Wuhan, China) and a rabbit polyclonal Vimentin antibody (10366-1-AP, ProteinTech Group, Inc., Wuhan, China) were added to slides, and they were incubated overnight at 4°C. Slides were washed with PBS, which were followed by incubation with Alexa Fluor^®^ 555-conjugated secondary antibodies for 30 min at 37°C. Finally, cell nuclei were stained using DAPI. Images were obtained using a microscope with a digital camera (Olympus, Tokyo, Japan).

### *In vivo* Xenograft Tumor Models

To assess the function of K17 *in vivo*, stable K17-silenced SW1990 cells (LV-K17 RNAi, 1 × 10^6^ cells in 100 μl of sterilized PBS) and stable scramble control SW1990 cells (LV-NC, 1 × 10^6^ cells in 100 μl of sterilized PBS) were injected into the left and right dorsal flanks of 4–weeks-old BALB/c male nude mice (Animal Center of the Chinese Academy of Science, Shanghai, China), respectively. Tumor sizes (length and width) were measured every week using calipers. Tumor volume was calculated as follows: tumor volume = (length × width^2^)/2. After 5 weeks, the mice were executed humanely, and the tumors were resected and photographed. This study was approved by the Research Ethics Committee of the Second Affiliated Hospital of Chongqing Medical University (China).

### Statistical Analysis

All analyses were conducted using SPSS 20.0 statistical software (IBM Corporation, Armonk, NY, USA). All data were expressed as the mean ± SD. Student's *t*-tests or one-way ANOVA were used to evaluate the differences. Paired Student's *t*-tests were applied to compare K17 expression in pancreatic cancer and paired adjacent non-cancerous tissues. The association of K17 expression with clinicopathologic features was analyzed by Pearson χ2 tests. Survival analysis was assessed by Kaplan Meier plots and log-rank tests. Independent prognostic factors were recognized through univariate and multivariate Cox proportional hazard regression models. Differences were considered statistically significant at *P* < 0.05.

## Results

### K17 mRNA Expression Was Upregulated in Pancreatic Cancer

**Figure 1:**
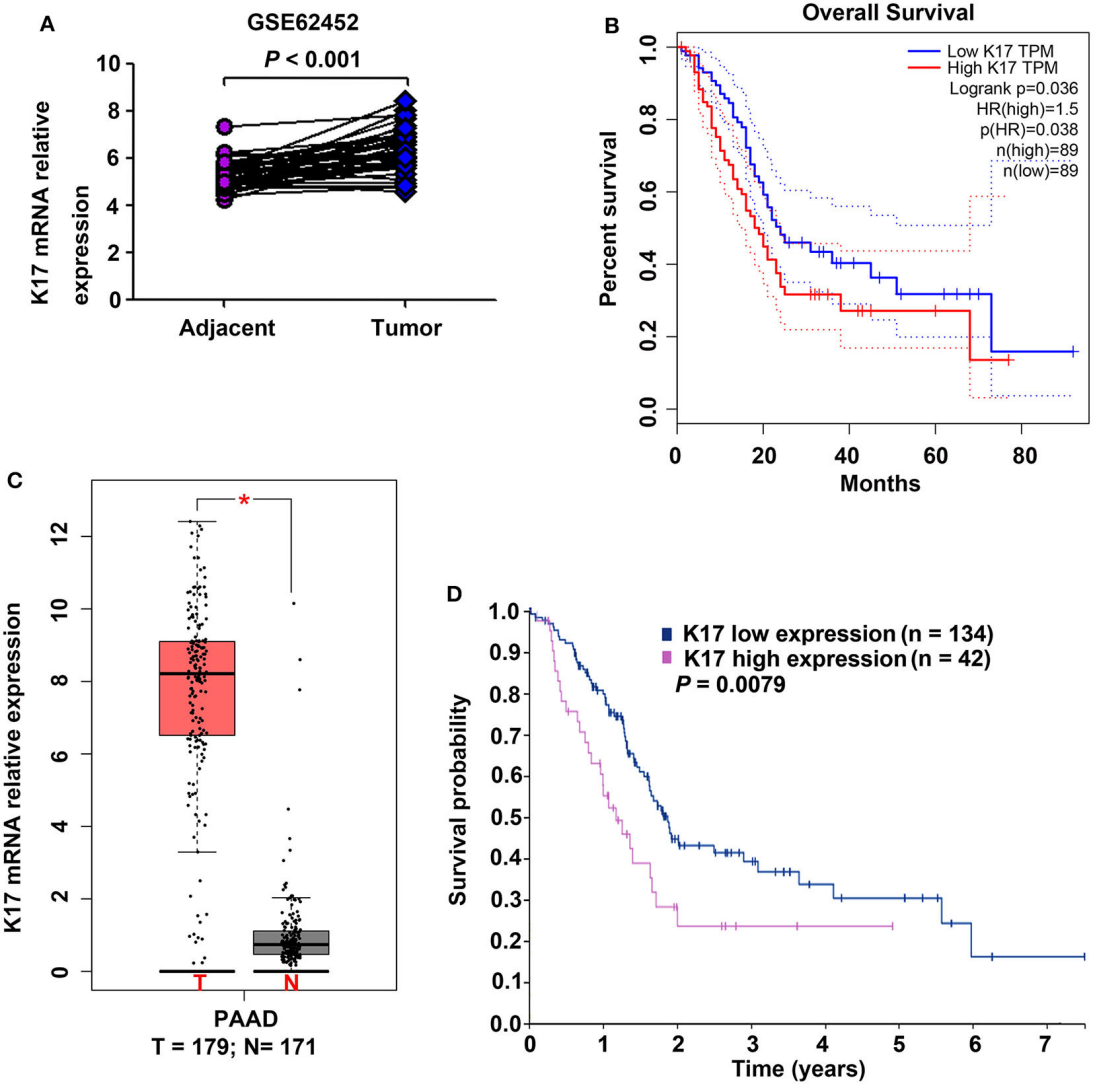
The expression of K17 mRNA and its correlation with overall survival in pancreatic cancer. **(A)** K17 mRNA expression in human pancreatic cancer tissues and matched adjacent normal tissues (*n* = 45 pairs) from a GEO cDNA microarray database (series GSE62452) was analyzed. **(B)** K17 mRNA expression in human pancreatic cancer tissues (T) and adjacent normal tissues (N) was analyzed by gene expression profiling interactive analysis (GEPIA) **(C,D)** The relationship between K17 mRNA expression and overall survival of pancreatic cancer patients was analyzed using the GEPIA database **(C)** and the Human Protein Atlas (www.proteinatlas.org/) **(D)**. ^*^P < 0.05.

To determine the expression of K17 mRNA in pancreatic cancer, we analyzed a GEO cDNA microarray database (series GSE62452) (Figure 1A) (18). As shown in Figure 1A, K17mRNA expression was markedly higher in pancreatic cancer samples than it was in adjacent normal tissues (*P* < 0.001). In addition, the results from the GEPIA database showed that the expression of K17mRNA was upregulated in pancreatic cancer samples compared that of normal tissues (*P* < 0.05, Figure 1B), and a high K17 mRNA level was significantly correlated with worse overall survival (*P* = 0.036, Figure 1C) (19). Moreover, Kaplan-Meier plots summarizing the results from the Human Protein Atlas (www.proteinatlas.org/) showed that patients with high K17 mRNA levels hah shorter overall survival (*P* = 0.0079, Figure 1D) (20).

### Overexpression of K17 Protein Correlated With Poor Prognosis of Pancreatic Cancer

**Figure 2:**
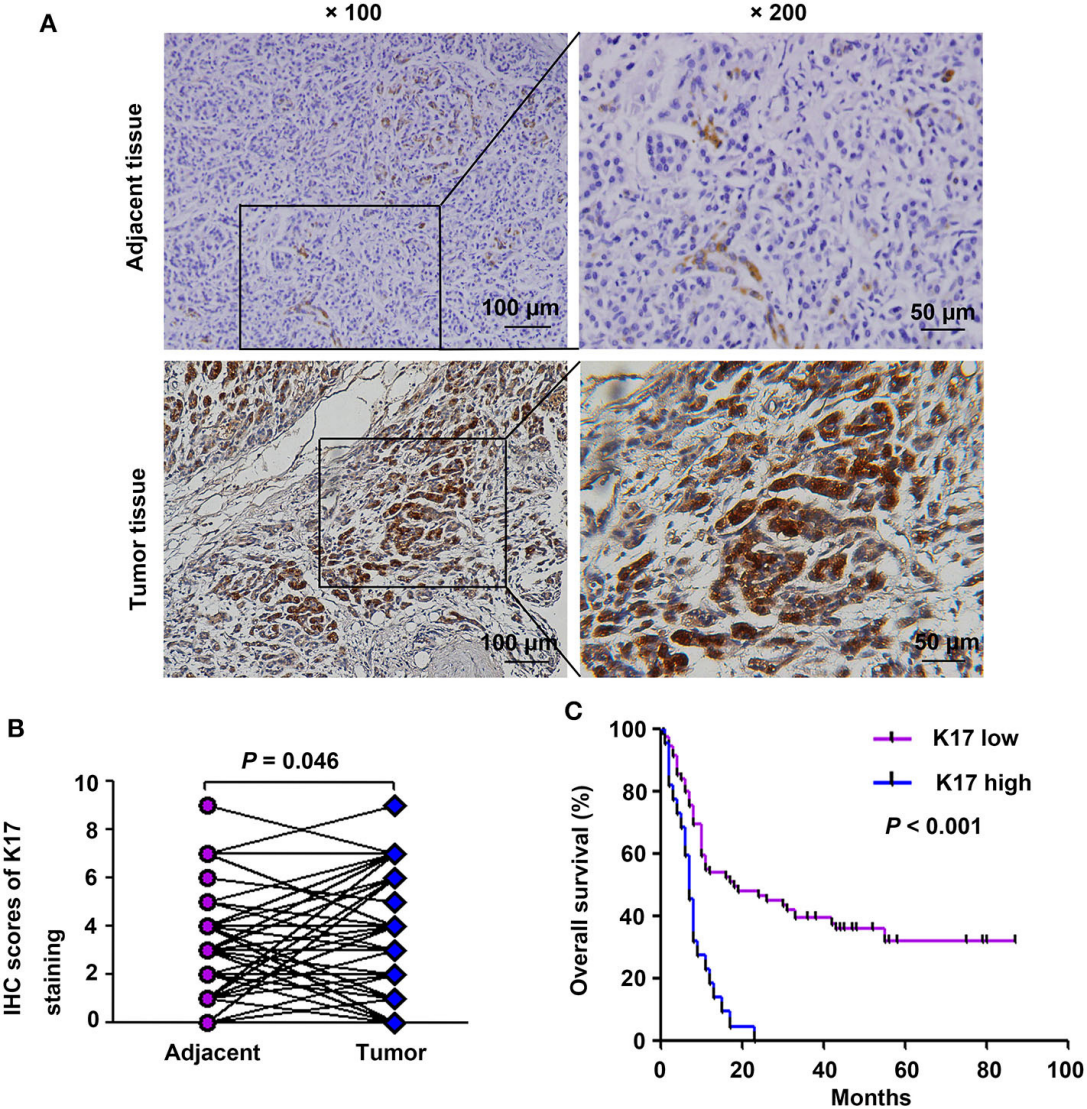
K17 protein expression was associated with poor clinical outcome in pancreatic cancer patients. **(A)** Representative immunohistochemical staining of K17 in pancreatic cancer tissues and adjacent normal tissues; × 100, scale bar = 100 μm; × 200, scale bar = 50 μm. **(B)** Comparison of total scores of K17 protein in pancreatic cancer tissues and their matched adjacent normal pancreatic tissues (*n* = 51 pairs). **(C)** The overall survival rate of patients with pancreatic cancer was evaluated based on K17 expression and determined using Kaplan-Meier analysis (*n* = 94).

To analyze the correlation between K17 protein expression levels and clinicpathological parameters, IHC assays were performed. Our results showed that K17 protein levels were increased in pancreatic cancer tissues compared with that of their matched adjacent normal pancreatic tissues (Figures 2A,B). In addition, we showed that K17 upregulation was significantly correlated with thepathological grade (*P* = 0.005) (Table 1). However, there was no significant correlation between K17 protein expression and age, gender, tumor size, TNM stage, tumor stage, lymph node metastasis or distant metastasis(*P* > 0.005) (Table 1). Moreover, univariate analysis indicated that the pathological grade and upregulated K17 expressionwere associated with overall survival (*P* = 0.002 and *P* = 0.000, respectively) (Table 2). Multivariate analysis showed that the pathological grade, lymph node metastasis and upregulated K17 expression were independent prognostic factors for overall survival (*P* = 0.002, *P* = 0.002 and *P* = 0.000, respectively) (Table 2). Kaplan–Meier analysis showed that pancreatic cancer patients with highK17 protein levels had shorter survival than patients with low K17 protein levels (*P* < 0.001, Figure 1C).

**Table 2:** Cox proportional hazard models for prognostic factors.

	**Univariate analysis**		**Multivariate analysis**	
	**HR (95% CI)**	***P-*value**	**HR (95% CI)**	***P-*value**
Age (> 60 vs. ≤ 60)	1.247 (0.756–2.057)	0.388		
Gender (female vs. male)	1.282 (0.761–2.159)	0.351		
Tumor size (≥4 vs. <4)	0.729 (0.430–1.235)	0.240		
Pathological grading (III + IV vs. I + II)	2.494 (1.384–4.495)	0.002^**^	2.441 (1.372–4.343)	0.002^**^
TNM stage (IIB–IV vs. I–IIA)	0.642 (0.121–3.411)	0.603		
Tumor stage (T3 + T4 vs. T1 + T2)	1.092 (0.578–2.063)	0.787		
Lymph node metastasis (positive vs. negative)	3.520 (0.648–19.130)	0.145	2.222 (1.340–3.685)	0.002^**^
Distant metastasis (M1 vs. M0)	-	-		
K17 expression (high vs. low)	2.766 (1.576–4.855)	0.000^**^	2.650 (1.534–4.577)	0.000^*^

* *Statistically significant (P < 0.05)*;

** *Statistically significant (P < 0.01)*.

### K17 Protein Was Upregulated in Pancreatic Cancer Cell Lines

**Figure 3:**
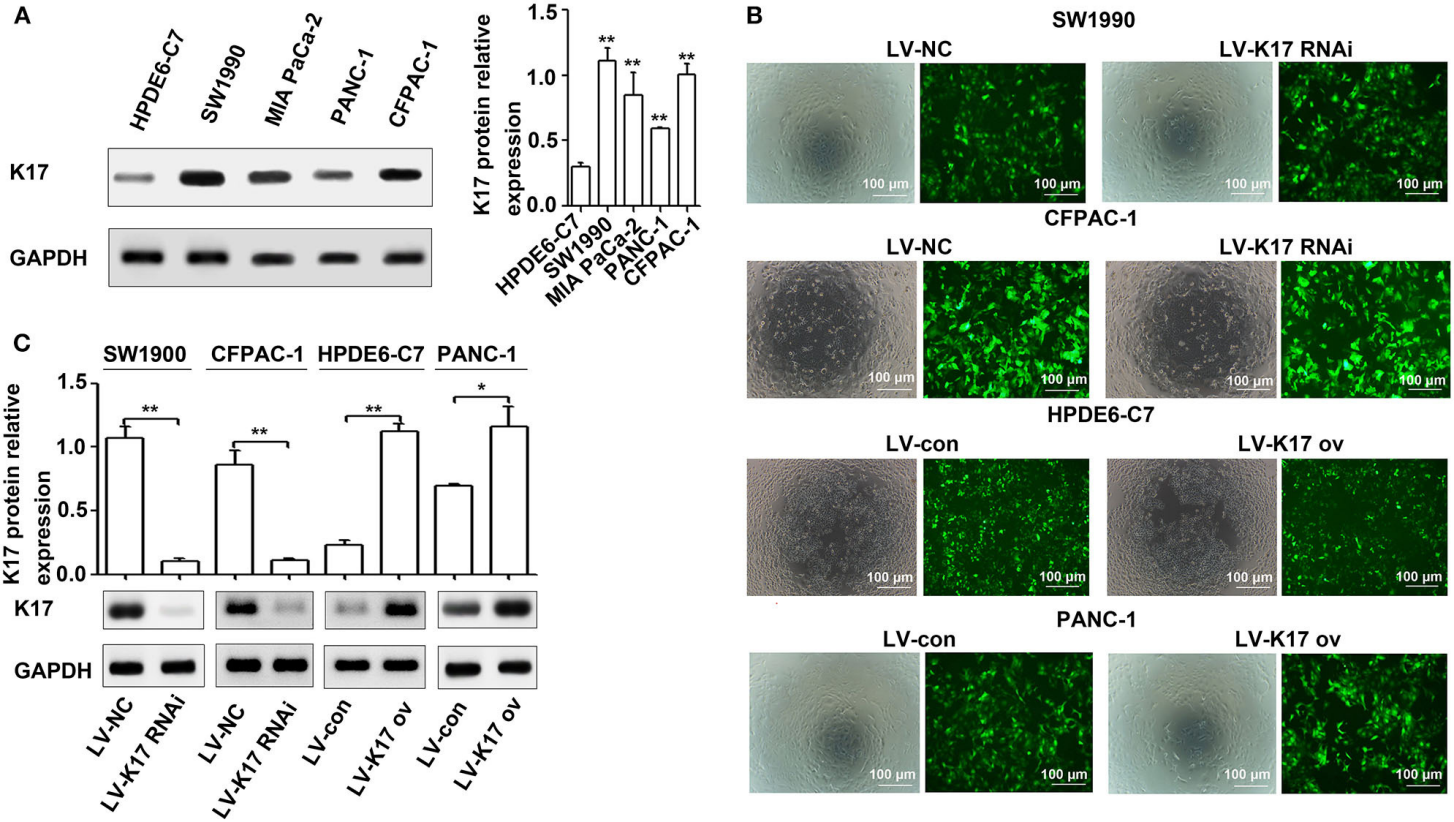
K17 protein was upregulated in pancreatic cancer cell lines. **(A)** The expression levels of K17 protein in four pancreatic cancer cell lines (CFPAC-1, MIA PaCa-2, PANC-1, and SW990) and one normal pancreatic cell line (HPDE6-C7) were determined by western blot analysis. **(B)** SW1990 and CFPAC-1 cells were infected with LV-K17 RNAi and LV-NC at an MOI of 20, and then were selected by puromycin treatment to establish stable K17 konckdown cell lines. HPDE6-C7 and PANC-1 cells were infected with LV-K17 ov and LV-con at an MOI of 30, and then were selected by puromycin treatment to establish stable K17 overexpressing cell lines. × 100, Scale Bar = 100 μm. **(C)** Western blot analysis was used to determine K17 protein expression in SW1990 and CFPAC-1 cells infected with LV-K17 RNAi and in HPDE6-C7 and PANC-1 cells infected with LV-K17. ^*^P < 0.05, and ^**^P < 0.01.

The expression levels of K17 protein in four pancreatic cancer cell lines (CFPAC-1, MIA PaCa-2, PANC-1 and SW990) and one normal pancreatic cell line (HPDE6-C7) were determined by western blot analysis. The results showed that K17 protein expression was significantly increased in pancreatic cancer cell lines compared with that of HPDE6-C7 cells (*P* < 0.01, Figure 3A). Then, SW1990 and CFPAC-1 cells were infected with LV-K17 RNAi and LV-NC at an MOI of 20, and then were selected by puromycin treatment to establish stable K17 konckdown cell lines (Figure 3B). HPDE6-C7 and PANC-1 cells were infected with LV-K17 ov and LV-con at an MOI of 30, and then were selected by puromycin treatment to establish stable K17 overexpressing cell lines (Figure 3B). As shown in Figure 3C, K17 protein expression in SW1990 and CFPAC-1 cells infected with LV-K17 RNAi was significantly decreased. Compared with the LV-con group, K17 protein expression in HPDE6-C7 and PANC-1 cells infected with LV-K17 ov was significantly increased (Figure 3C).

### K17 Repressed Pancreatic Cancer Cell Growth *in vitro* and *in vivo*

**Figure 4:**
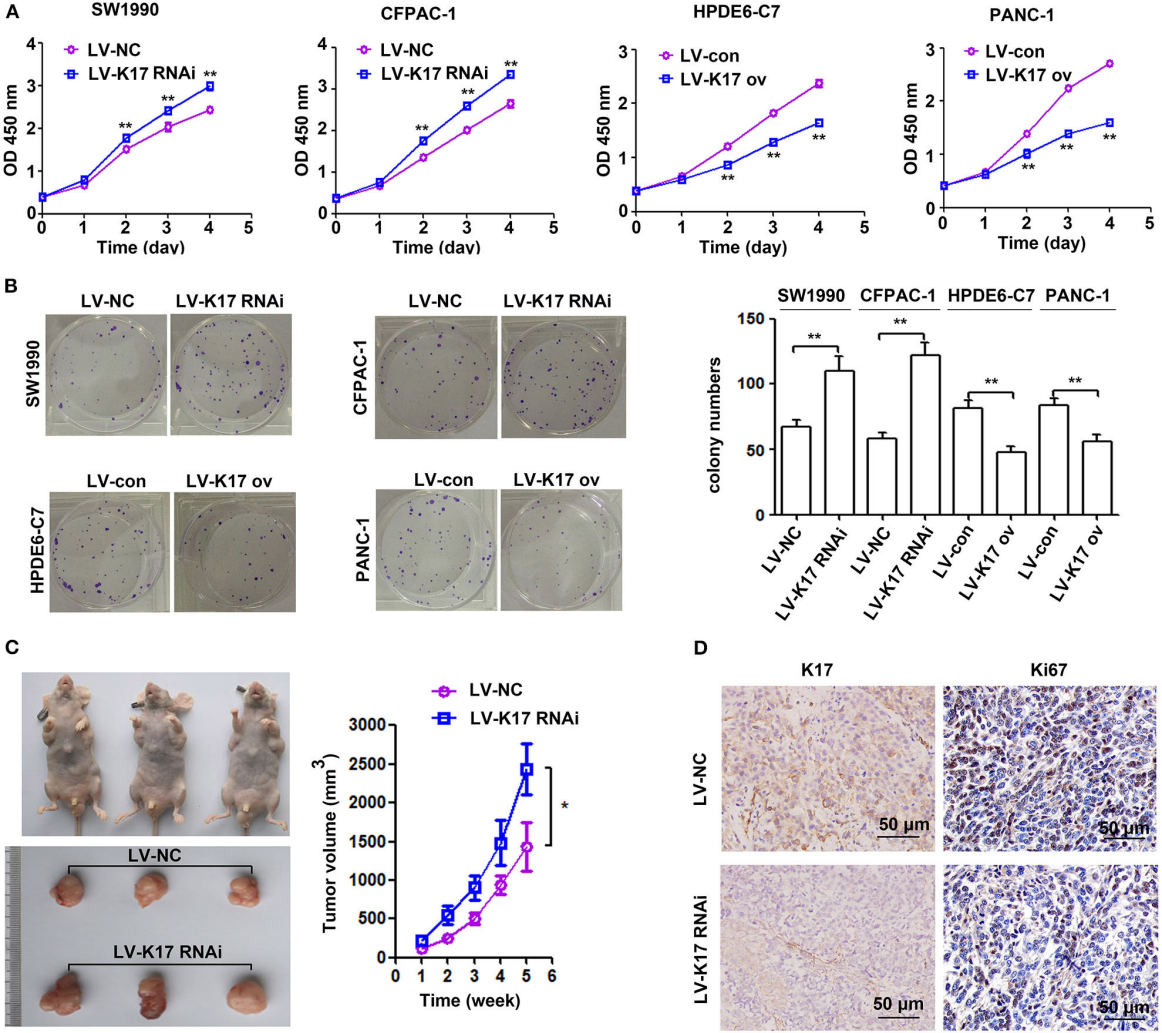
K17 inhibited pancreatic cancer cell growth *in vitro* and *in vivo*. **(A,B)** The proliferation ability of stable K17 silenced SW1990 and CFPAC-1 cells and stable K17 upregulated HPDE6-C7 and PANC-1 cells was determined by CCK-8 assays **(A)** and colony formation assays **(B)**. **(C)** K17 inhibition significantly elevated tumor growth yielding larger tumor volumes than those of the LV-NC group. **(D)** Ki67 IHC analysis of tumors from nude mice. × 200 Scale Bar = 50 μm. ^*^P < 0.05, and ^**^P < 0.01.

To determine the effect of K17 on pancreatic cancer cell proliferation, CCK-8 and colony formation assays were performed. As shown in Figure 4A, K17 inhibition significantly elevated cell proliferation ability, while K17 upregulation significantly repressed cell proliferation ability. A similar trend was observed in colony formation assays (Figure 4B). To confirm the role of K17 *in vivo*, xenograft tumor models were used. Our results showed that K17 inhibition significantly increased tumor growth, producing larger tumor volumes than what was observed in the LV-NC group (Figure 4C). In addition, more Ki67-positive cells were found in the LV-K17 RNAi group than in the LV-NC group (Figure 4D).

### K17 Induced Cell Cycle Arrest and Cell Apoptosis in Pancreatic Cancer Cells *in vitro*

**Figure 5:**
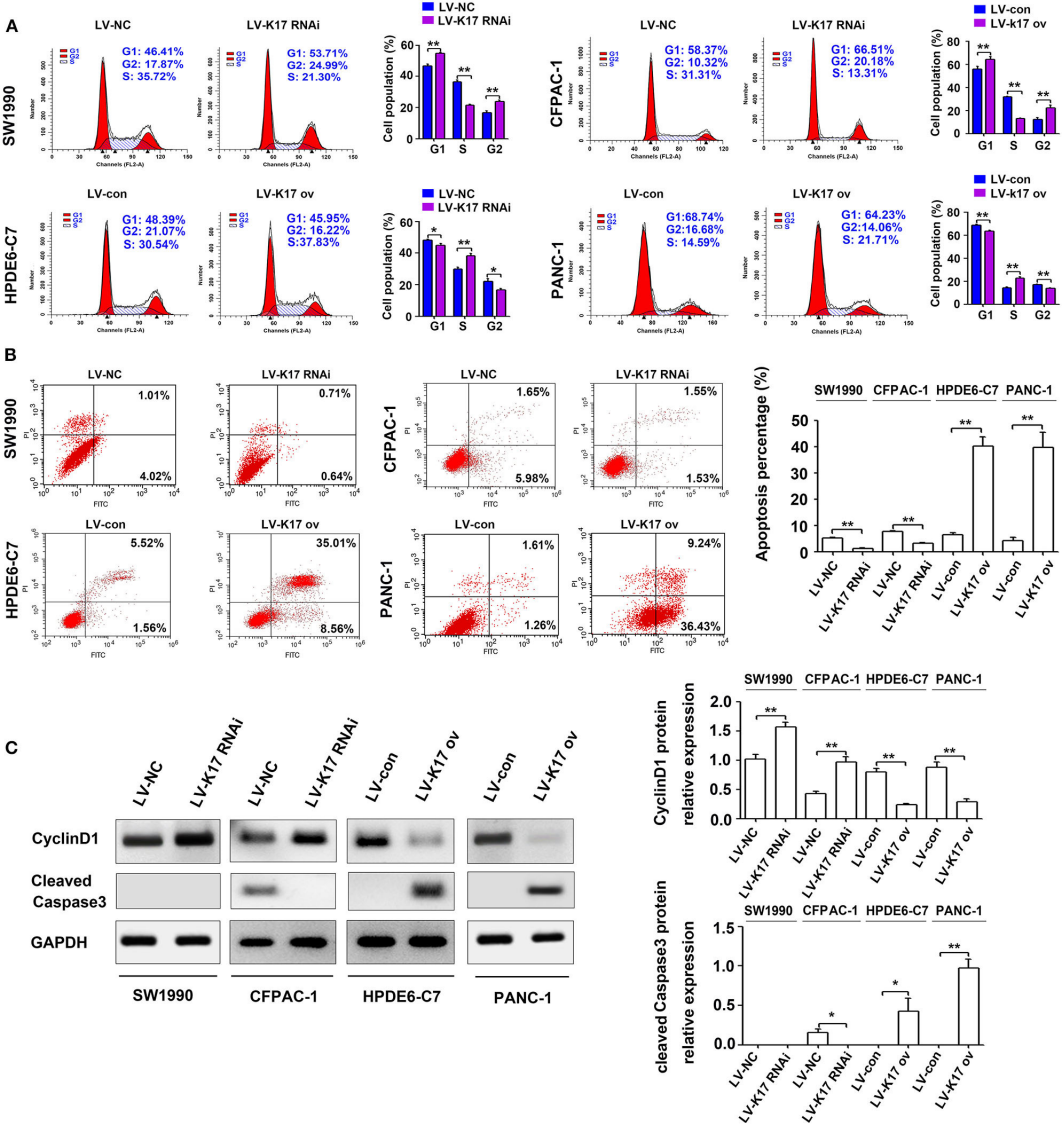
K17 induced cell cycle arrest and cell apoptosis in pancreatic cancer cells *in vitro*. **(A)** Cell cycle distribution was analyzed by flow cytometry in stable K17 silenced SW1990 and CFPAC-1 cells and stable K17 upregulated HPDE6-C7 and PANC-1 cells. **(B)** Flow cytometry was used to determine the percentage of apoptotic cells in stable K17-silenced SW1990 and CFPAC-1 cells and in stable K17 upregulated HPDE6-C7 and PANC-1 cells. **(C)** Western blot analysis was performed to determine the expression of CyclinD1 and cleaved Caspase3 protein in stable K17-silenced SW1990 and CFPAC-1 cells and in stable K17 upregulated HPDE6-C7 and PANC-1 cells. ^*^P < 0.05, and ^**^P < 0.01.

As shown in Figure 5A, knocking down K17 in SW1990 and CFPAC-1 cells increased the number of cells in G1 and G2 phase and decreased the number of cells in S phase; however, K17 upregulation in HPDE6-C7 and PANC-1 cells decreased the number of cells in G1 and G2 phase and increased the number of cells in S phase. In addition, we explored the role of K17 in the apoptosis of SW1990, CFPAC-1, HPDE6-C7, and PANC-1 cells. Our results showed that downregulating K17 in SW1990 and CFPAC-1 cells resulted in a significant decrease in apoptotic cell death, while K17 overexpression in HPDE6-C7 and PANC-1 cells caused an increase in apoptotic cell death (Figure 5B). Because an inhibition of proliferation was observed in HPDE6-C7 and PANC-1 cells after K17 upregulation, the associated cell cycle regulators and apoptosis markers were analyzed by western blot. As shown in Figure 5C, increased CyclinD1 was observed in LV-K17 RNAi infected SW1990 and CFPAC-1 cells, while decreased CyclinD1 was observed in LV-K17 ov infected HPDE6-C7 and PANC-1 cells. In addition, overexpression of K17 significantly induced the expression of cleaved Caspase3, while there was no difference between LV-K17 RNAi infected SW1990 cells and LV-NC infected SW1990 cells (Figure 5C).

### K17 Inhibited Cell Migration and Invasion in Pancreatic Cancer Cells *in vitro*

**Figure 6:**
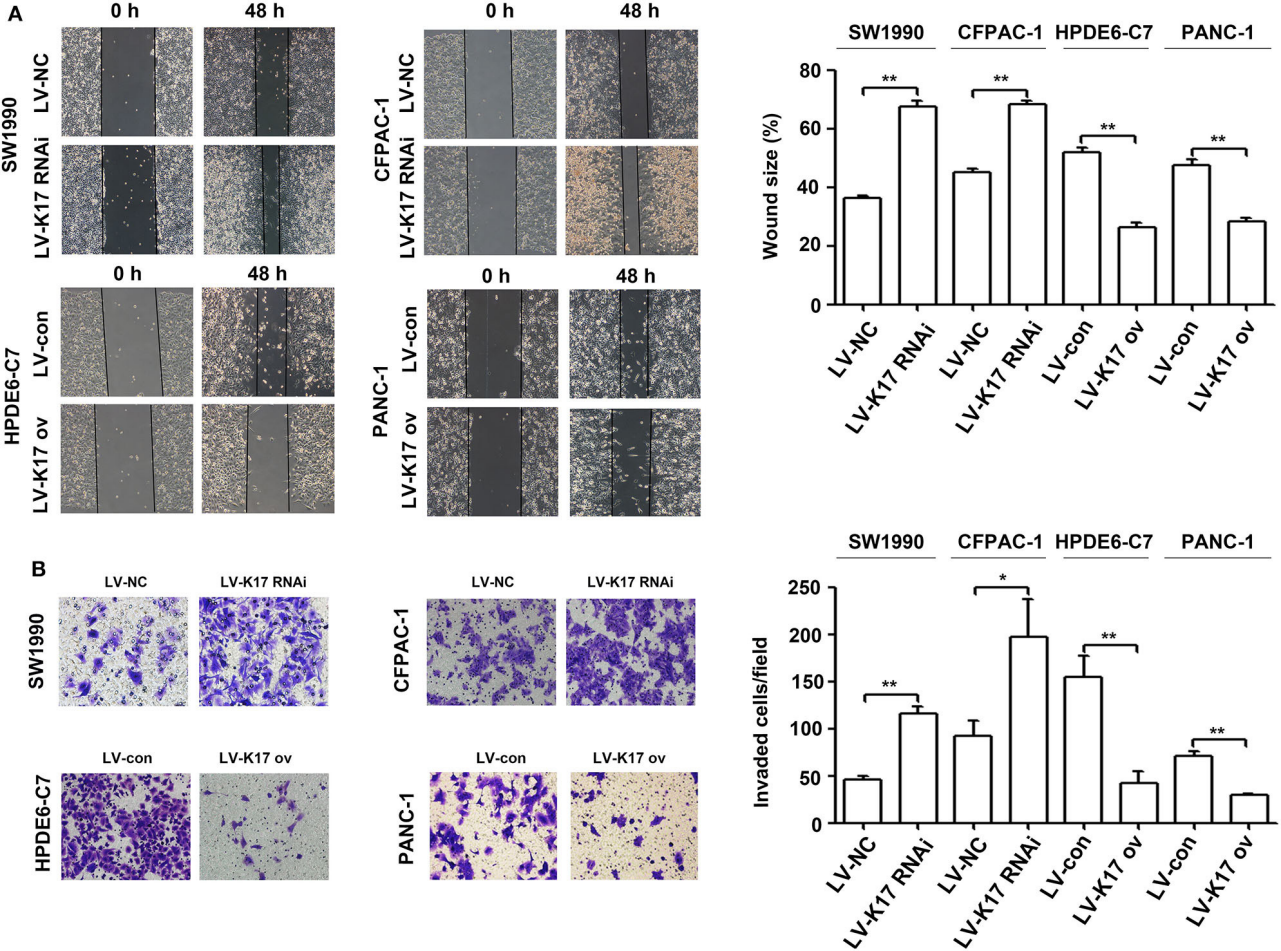
K17 inhibited cell migration and invasion in pancreatic cancer cells *in vitro*. **(A)** A wound healing assay was used to determine the cell migration ability of stable K17-silenced SW1990 and CFPAC-1 cells and of stable K17 upregulated HPDE6-C7 and PANC-1 cells. **(B)** Cell invasion of stable K17-silenced SW1990 and CFPAC-1 cells and stable K17 upregulated HPDE6-C7 and PANC-1 cells was tested with a Transwell assay. ^*^P < 0.05, and ^**^P < 0.01.

To determine whether K17 regulated pancreatic cancer cell migration and invasion, wound healing and Transwell invasion experiments were performed. As shown in Figure 6A, the migration distance of SW1990 and CFPAC-1 cells in the LV-K17 RNAi group was significantly larger than that of the LV-NC group, while migration distance of HPDE6-C7 and PANC-1 cells in the LV-K17 ov group was significantly lower than that of the LV-con group. Furthermore, the results from the Transwell invasion experiment demonstrated that the number of invasive SW1990 and CFPAC-1 cells in the LV-K17 RNAi group was significantly greater than the number in the LV-NC group, while the number of invasive HPDE6-C7 and PANC-1 cells in the LV-K17 ov group was significantly less than those in the LV-con group (Figure 6B).

### K17 Inhibited the Epithelial-Mesenchymal Transition (EMT) in Pancreatic Cancer Cells

**Figure 7:**
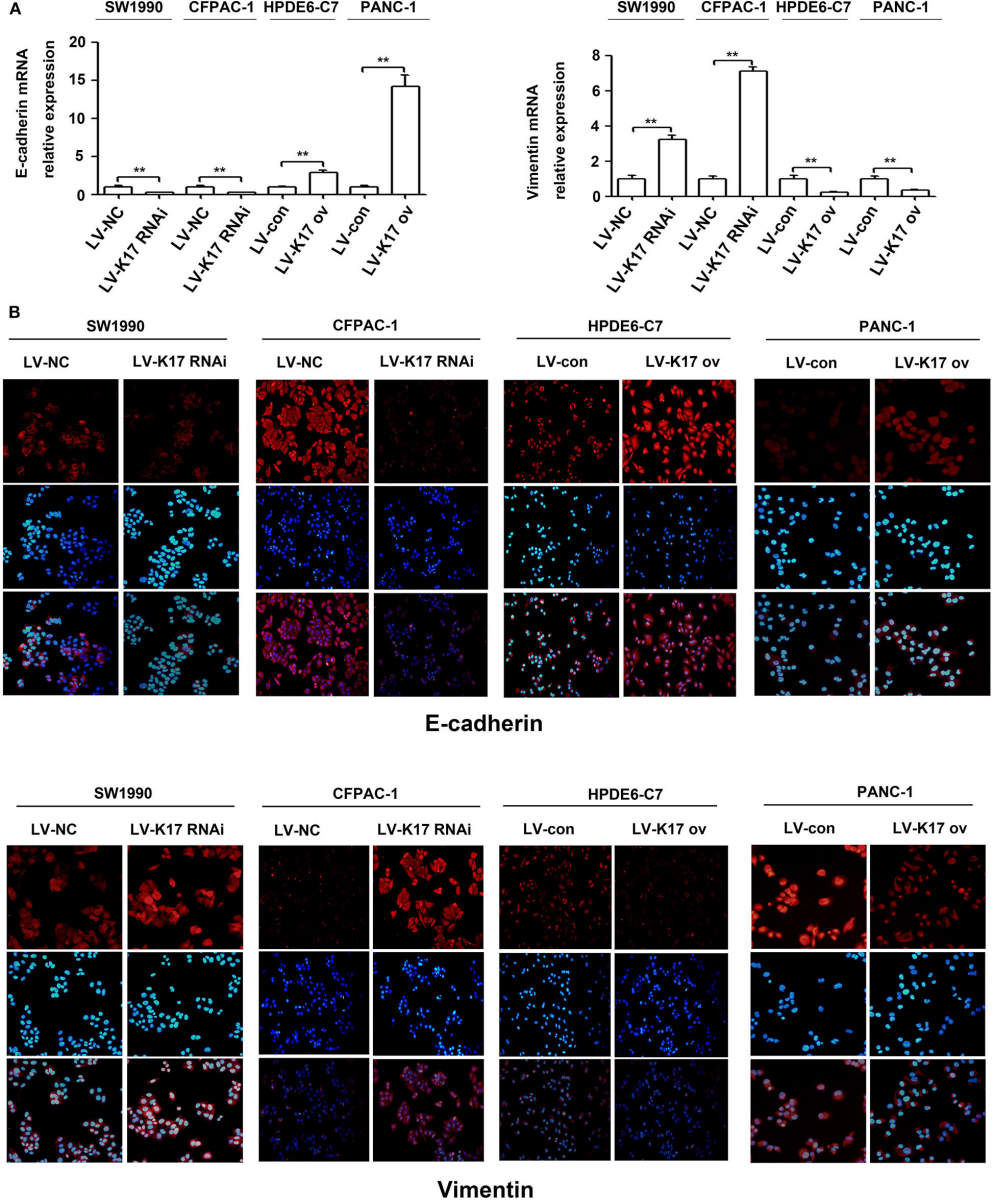
K17 inhibited the EMT in pancreatic cancer cells. **(A)** qRT-PCR analysis of E-cadherin and Vimentin mRNA expression in stable K17-silenced SW1990 and CFPAC-1 cells and in stable K17 upregulated HPDE6-C7 and PANC-1 cells. **(B)** Immunofluorescence staining of E-cadherin and Vimentin protein expression in stable K17-silenced SW1990 and CFPAC-1 cells and in stable K17 upregulated HPDE6-C7 and PANC-1 cells. ^**^P < 0.01.

Epithelial-mesenchymal transition (EMT) plays an important role in *in situ* infiltration and distant metastasis of many types of cancer, including pancreatic cancer (21). To further investigate the molecular mechanism mediating the aggressive effects of K17 on pancreatic cancer cells, we determined whether K17 regulated EMT in pancreatic cancer cells. As shown in Figure 7A, knocking down K17 significantly repressed the expression levels of E-cadherin mRNA, and induced the expression levels of Vimentin mRNA. However, K17 upregulation significantly increased the expression levels of E-cadherin mRNA, and decreased the expression levels of Vimentin mRNA (Figure 7A). A similar trend was observed in immunofluorescence assays (Figure 7B).

## Discussion

The present study showed that K17 was significantly upregulated in pancreatic cancer, and elevated K17 expression was an independent adverse prognostic factor for the overall survival of patients with pancreatic cancer. In addition, we initially found that K17 knockdown induced pancreatic cancer cell proliferation, migration, invasion and EMT, and K17 overexpression inhibited pancreatic cancer cell proliferation, migration, invasion, and EMT. Thus, K17 functions as a potential tumor suppressor, even though it is upregulated in pancreatic cancer.

Upregulated K17 has been reported in many human cancers, including oral cancer, non-small cell lung cancer, basal cell carcinoma and cervical cancer, as well as in pancreatic cancer (8–14). In addition, Roa-Peña et al. demonstrated that increased K17 protein expression is associated with decreased survival of patients with PDAC (14). In this study, we analyzed K17 gene expression in two published databases (a GEO cDNA microarray database and a GEPIA database). Our results showed that K17 gene expression was significantly upregulated in pancreatic cancer (Figures 1A,B). Furthermore, the results from the GEPIA database and from Human Protein Atlas analyses indicated that upregulated K17 mRNA levels were associated with a poor prognosis in pancreatic cancer (Figures 1C,D). IHC analysis showed that K17 protein levels were increased in pancreatic cancer tissues compared with that in their matched adjacent normal pancreatic tissues (Figures 2A,B), and upregulated K17 protein levels were significantly correlated with pathological grade and shorter overall survival (Figure 2C and Table 1). Thus, K17 may be a useful prognostic factor for patients with pancreatic cancer.

Previous studies have revealed that K17 promotes tumor progression in numerous cancer types, including gastric cancer, non-small cell lung cancer, oral squamous cell carcinomas and Ewing sarcoma (8, 9, 22–25). For example, K17 induced tumor growth and invasion in gastric cancer (22). In oral cancer, K17 induced cell proliferation and migration by stimulating the Akt/mTOR pathway and promoting glucose uptake (8). In addition, K17 was involved in GLI-mediated oncogenic transformation and cellular adhesion in Ewing sarcoma (25). In non-small cell lung cancer, K17 promoted cell proliferation, invasion and EMT (9). Thus, these findings imply that K17 has an oncogenic role in tumor progression. Based on recent reports and our above results, we hypothesize that K17 functions as an oncogene in pancreatic cancer. In contrast, our functional studies first showed that K17 knockdown induced pancreatic cancer cell proliferation and colony formation (Figures 4A,B). However, K17 upregulation inhibited pancreatic cancer cell proliferation and colony formation (Figures 4A,B). Next, to determine whether the suppression of cell proliferation was due to the inhibition of the cell cycle or the increased apoptosis by K17, cell cycle and apoptosis assays were performed *in vitro* (Figure 5). Our results showed that knocking down K17 accelerated the cell cycle and decreased cell apoptosis, while K17 overexpression caused cell cycle arrest and cell apoptosis (Figure 5). Meanwhile, we also found that knocking down K17 induced pancreatic cancer cell migration and invasion, while overexpressing K17 repressed cell migration and invasion (Figure 6). EMT, a well-characterized embryological process, is one of the mechanisms by which cancer cells acquire metastatic potential through gaining enhanced mobility and invasiveness (26, 27). Our results showed that K17 knockdown induced EMT through reducing E-cadherin expression and increasing Vimentin expression (Figure 7). However, K17 upregulation repressed EMT through increasing E-cadherin expression and decreasing Vimentin expression (Figure 7). Thus, the anti-migration and invasion function of K17 may be mediated by suppressing EMT. These results suggested that elevated K17 may play a tumor suppressor function in pancreatic cancer.

It has been reported that several tumor suppressors are overexpressed in cancer (28, 29). For example, p16^lnk4a^ was upregulated in many human tumors and functioned as a tumor suppressor (28). Accumulating evidence shows that oncogene-induced senescence can occur in response to oncogenic insults and is considered an important tumor suppressor mechanism (30). Interestingly, p16^lnk4a^-mediated senescent-like arrest was reported in response to oncogenic Ras mediated stimulation in normal primary cells (31). In addition, p16^lnk4a^ was upregulated in human naevi, and elevated p16^lnk4a^ was involved in senescence-associated growth arrest, protecting the cell from malignant transformation (32, 33). This pattern of p16^Ink4a^ overexpression suggests that p16^Ink4a^ inhibition is the main step in bypassing senescence. Montes et al. (33) found that p16 ^lnk4a^ was mutated or homozygously deleted in ~40% of melanoma cases. However, we found that K17 was upregulated in pancreatic cancer when compared with matched adjacent normal pancreatic tissues. Thus, our results suggest that other mechanisms are needed to induce K17 upregulation in pancreatic cancer. A recent study showed that oncogene was also downregulated in cancer (34). Ma et al. (34) found that carbonic anhydrase-related protein VIII (CA8) was downregulated in renal cell carcinoma and that CA8 upregulation induced cell proliferation and migration. Thus, we speculate that the low expression or loss of activity of oncogenes such as CA8 is one of the reasons for the high expression of tumor suppressor genes such as K17, GATA4 and p16^lnk4a^.

**Figure 8:**
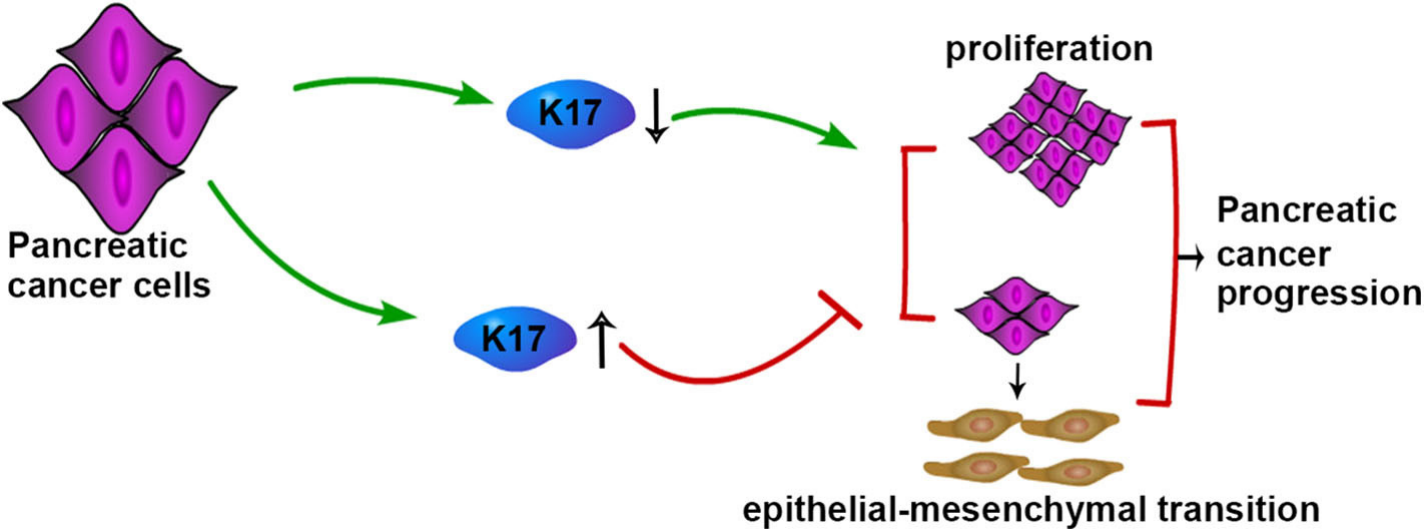
Schematic model of K17-regulated pancreatic cancer progression. K17 up-regulation inhibits the proliferation and EMT of pancreatic cancer cells, while K17 down-regulation promotes the proliferation and EMT of pancreatic cancer cells.

In conclusion, our study showed that K17 was upregulated in pancreatic cancer, and upregulated K17 was associated with a poorer outcome in patients with pancreatic cancer. In addition, we initially found that K17 inhibited pancreatic cancer cell proliferation, migration, invasion and EMT, although it was upregulated in pancreatic cancer (Figure 8).

## Data Availability Statement

All datasets presented in this study are included in the article/Supplementary Material.

## Ethics Statement

The studies involving human participants were reviewed and approved by the Research Ethics Committee of the Second Affiliated Hospital of Chongqing Medical University (China). The patients/participants provided their written informed consent to participate in this study.

## Author Contributions

YZ, MZ, and YY performed research and wrote the first draft. JG collected and analyzed the data. YY is the guarantor. All authors contributed to the design and interpretation of the study and to further drafts.

## Conflict of Interest

The authors declare that the research was conducted in the absence of any commercial or financial relationships that could be construed as a potential conflict of interest.

## References

[1] Goral V. Pancreatic cancer: pathogenesis and diagnosis. Asian Pac J Cancer Prev. (2015) 16:5619–24. 10.7314/APJCP.2015.16.14.5619 26320426

[2] Zhang L Sanagapalli S Stoita A. Challenges in diagnosis of pancreatic cancer. World J Gastroenterol. (2018) 24:2047–60. 10.3748/wjg.v24.i19.2047 29785074 PMC5960811

[3] Zhu H Li T Du Y Li M. Pancreatic cancer: challenges and opportunities. BMC Med. (2018) 16:214. 10.1186/s12916-018-1215-3 30463539 PMC6249728

[4] Zhang X Yin M Zhang LJ. Keratin 6, 16 and 17-critical barrier alarmin molecules in skin wounds and psoriasis. Cells. (2019) 8:807. 10.3390/cells8080807 31374826 PMC6721482

[5] Schweizer J Bowden PE Coulombe PA Langbein L Lane EB Magin TM. New consensusnomenclature for mammalian keratins. J Cell Biol. (2006) 174:169–74. 10.1083/jcb.200603161 16831889 PMC2064177

[6] Troyanovsky SM Leube RE Franke WW. Characterization of the human gene encoding cytokeratin 17 and its expression pattern. Eur J Cell Biol. (1992) 59:127–37. 1281771

[7] Kim S Wong P Coulombe PA. A keratin cytoskeletal protein regulates protein synthesis and epithelial cell growth. Nature. (2006) 441:362–5. 10.1038/nature04659 16710422

[8] Khanom R Nguyen CT Kayamori K Zhao X Morita K Miki Y. Keratin 17 is induced in oral cancer and facilitates tumor growth. PLoS ONE. (2016) 11:e0161163. 10.1371/journal.pone.0161163 27512993 PMC4981360

[9] Wang Z Yang MQ Lei L Fei LR Zheng YW Huang WJ. Overexpression of KRT17 promotes proliferation and invasion of non-small cell lung cancer and indicates poor prognosis. Cancer Manag Res. (2019) 11:7485–97. 10.2147/CMAR.S218926 31496806 PMC6689799

[10] Depianto D Kerns ML Dlugosz AA Coulombe PA. Keratin17 promotes epithelial proliferation and tumor growth by polarizing the immune response in skin. Nat Genet. (2010) 42:910–4. 10.1038/ng.665 20871598 PMC2947596

[11] Escobar-Hoyos LF Yang J Zhu J Cavallo JA Zhai H Burke S. Keratin 17 in premalignant and malignant squamous lesions of the cervix: proteomic discovery and immunohistochemical validation as a diagnostic and prognostic biomarker. Mod Pathol. (2014) 27:621–30. 10.1038/modpathol.2013.166 24051697 PMC4026928

[12] Iacobuzio-Donahue CA Maitra A Olsen M Lowe AW van Heek NT Rosty C. Exploration of global gene expression patterns in pancreatic adenocarcinoma using cDNA microarrays. Am. J. Pathol. (2003) 162:1151–62. 10.1016/S0002-9440(10)63911-9 12651607 PMC1851213

[13] Bournet B Pointreau A Souque A Oumouhou N Muscari F Lepage B. Gene expression signature of advanced pancreatic ductal adenocarcinoma using low density array on endoscopic ultrasound-guided fine needle aspiration samples. Pancreatology. (2012) 12:27–34. 10.1016/j.pan.2011.12.003 22487470

[14] Roa-Peña L Leiton CV Babu S Pan CH Vanner EA Akalin A. Keratin 17 identifies the most lethal molecular subtype of pancreatic cancer. Sci Rep. (2019) 9:11239. 10.1038/s41598-019-47519-4 31375762 PMC6677817

[15] Tian XP Jin XH Li M Huang WJ Xie D Zhang JX. The depletion of PinX1 involved in the tumorigenesis of non-small cell lung cancer promotes cell proliferation via p15/cyclin D1 pathway. Mol Cancer. (2017) 16:74. 10.1186/s12943-017-0637-4 28372542 PMC5379637

[16] Chen Y Peng C Chen J Chen D Yang B He B. WTAP facilitates progression of hepatocellular carcinoma via m6A-HuR-dependent epigenetic silencing of ETS1. Mol Cancer. (2019) 18:127. 10.1186/s12943-019-1053-8 31438961 PMC6704583

[17] Livak KJ Schmittgen TD. Analysis of relative gene expression data using real-time quantitative PCR and the 2^−ΔΔ*CT*^ Method. Methods. (2001) 25:402–8. 10.1006/meth.2001.1262 11846609

[18] Yang S He P Wang J Schetter A Tang W Funamizu N. A novel MIF signaling pathway drives the malignant character of pancreatic cancer by targeting NR3C2. Cancer Res. (2016) 76:3838–50. 10.1158/0008-5472.CAN-15-2841 27197190 PMC4930741

[19] Tang Z Li C Kang B Gao G Li C Zhang Z. GEPIA: a web server for cancer and normal gene expression profiling and interactive analyses. Nucleic Acids Res. (2017) 45:W98–102. 10.1093/nar/gkx247 28407145 PMC5570223

[20] Uhlen M Zhang C Lee S Sjöstedt E Fagerberg L Bidkhori G. A pathology atlas of the human cancer transcriptome. Science. (2017) 357:eaan2507. 10.1126/science.aan2507 28818916

[21] Rahib L Smith BD Aizenberg R Rosenzweig AB Fleshman JM Matrisian LM. Projecting cancer incidence and deaths to 2030: the unexpected burden of thyroid, liver, and pancreas cancers in the United States. Cancer Res. (2014) 74:2913–21. 10.1158/0008-5472.CAN-14-0155 24840647

[22] Chivu-Economescu M Dragu DL Necula LG Matei L Enciu AM Bleotu C. Knockdown of KRT17 by siRNA induces antitumoral effects on gastric cancer cells. Gastric Cancer. (2017) 20:948–59. 10.1007/s10120-017-0712-y 28299464

[23] Liu J Liu L Cao L Wen Q. Keratin 17 promotes lung adenocarcinoma progression by enhancing cell proliferation and invasion. Med Sci Monit. (2018) 24:4782–90. 10.12659/MSM.909350 29991674 PMC6069497

[24] Mikami Y Fujii S Nagata K Wada H Hasegawa K Abe M. GLI-mediated Keratin 17 expression promotes tumor cell growth through the anti-apoptotic function in oral squamous cell carcinomas. J Cancer Res *Clin Oncol*. (2017) 143:1381–93. 10.1007/s00432-017-2398-2 28342001 PMC11819195

[25] Sankar S Tanner JM Bell R Chaturvedi A Randall RL Beckerle MC. A novel role for keratin 17 in coordinating oncogenic transformation and cellular adhesion in Ewing sarcoma. Mol Cell Biol. (2013) 33:4448–60. 10.1128/MCB.00241-13 24043308 PMC3838177

[26] Yeung KT Yang J. Epithelial-mesenchymal transition in tumor metastasis. Mol Oncol. (2017) 11:28–39. 10.1002/1878-0261.12017 28085222 PMC5242415

[27] Puisieux A Brabletz T Caramel J. Oncogenic roles of EMT-inducing transcription factors. Nat Cell Biol. (2014) 16:488–94. 10.1038/ncb2976 24875735

[28] Chauhan S Sen S Sharma A Kashyap S Tandon R Bajaj MS. p16INK4a overexpression as a predictor of survival in ocular surface squamous neoplasia, Br. J Ophthalmol. (2018) 102:840–7. 10.1136/bjophthalmol-2017-311276 29511060

[29] Gong Y Zhang L Zhang A Chen X Gao P Zeng Q. GATA4 inhibits cell differentiation and proliferation in pancreatic cancer. PLoS ONE. (2018) 13:e0202449. 10.1371/journal.pone.0202449 30142155 PMC6108473

[30] Collado M Gil J Efeyan A Guerra C Schuhmacher AJ Barradas M. Tumour biology: senescence in premalignant tumours. Nature. (2005) 436:642. 10.1038/436642a 16079833

[31] Cánepa ET Scassa ME Ceruti JM Marazita MC Carcagno AL Sirkin PF. INK4 proteins, a family of mammalian CDK inhibitors with novel biological functions. IUBMB Life. (2007) 59:419–26. 10.1080/15216540701488358 17654117

[32] Michaloglou C Vredeveld LC Soengas MS Denoyelle C Kuilman T van der Horst CMAM. BRAFE600-associated senescence-like cell cycle arrest of human naevi. Nature. (2005) 436:720–4. 10.1038/nature03890 16079850

[33] Montes M Nielsen MM Maglieri G Jacobsen A Højfeldt J Agrawal-Singh S. The lncRNA MIR31HG regulates p16(INK4A) expression to modulate senescence. Nat Commun. (2015) 6:6967. 10.1038/ncomms7967 25908244

[34] Ma HL Yu SJ Chen J Ding XF Chen G Liang Y Pan JL. CA8 promotes RCC proliferation and migration though its expression level is lower in tumor compared to adjacent normal tissue. Biomed Pharmacother. (2020) 121:109578. 10.1016/j.biopha.2019.109578 31715371

