# Supplementary figures and images for: Keratin 17 Suppresses Cell Proliferation and Epithelial-Mesenchymal Transition in Pancreatic Cancer
Source: Front Med (Lausanne). 2020 Nov 26;7:572494. doi: 10.3389/fmed.2020.572494 (PMC7726264; doi:10.3389/fmed.2020.572494)

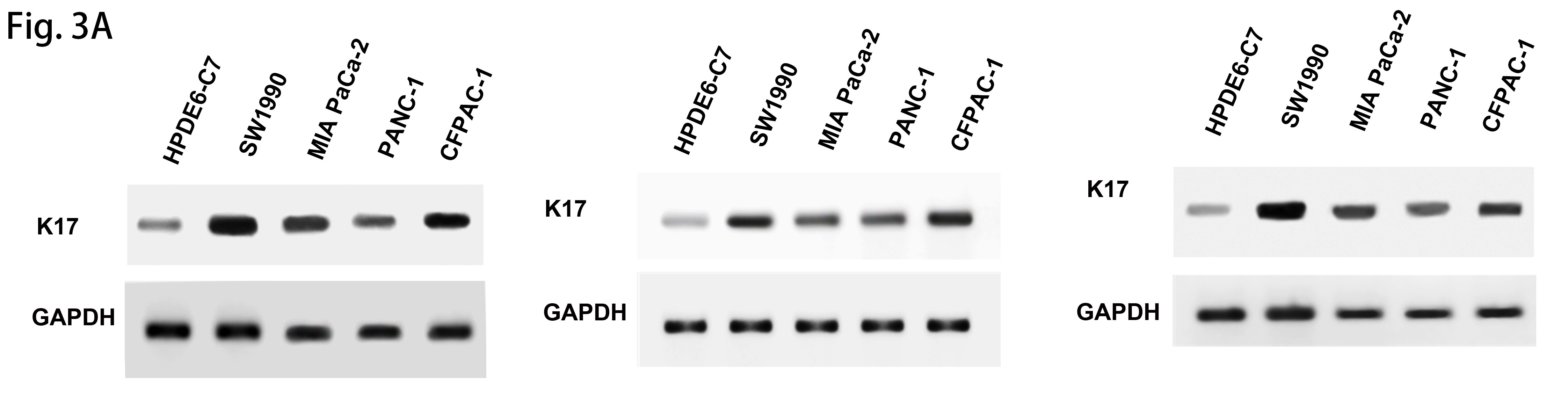

Supplement: Supplementary file 1 [file Image_1.JPEG]

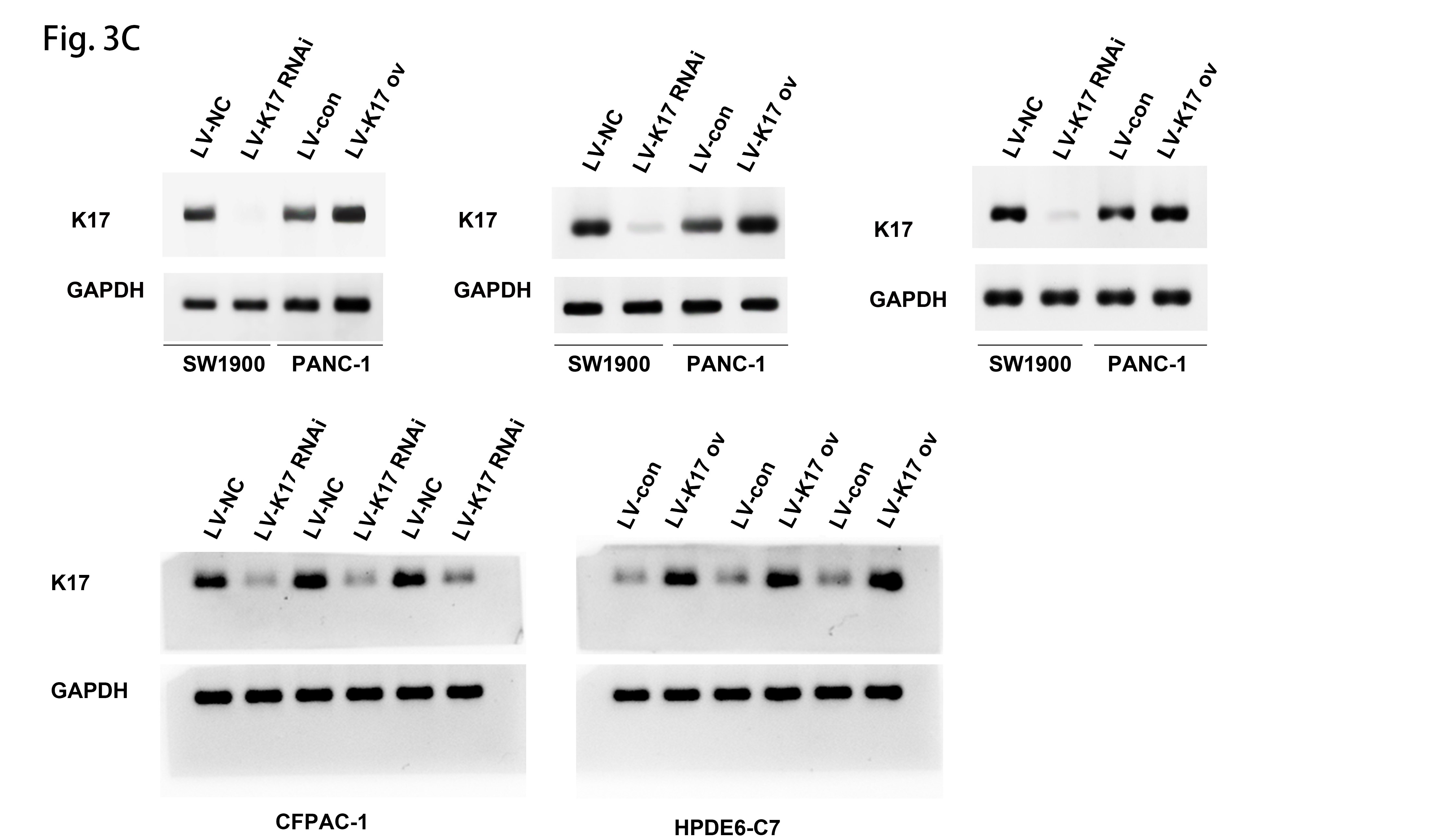

Supplement: Supplementary file 2 [file Image_2.JPEG]

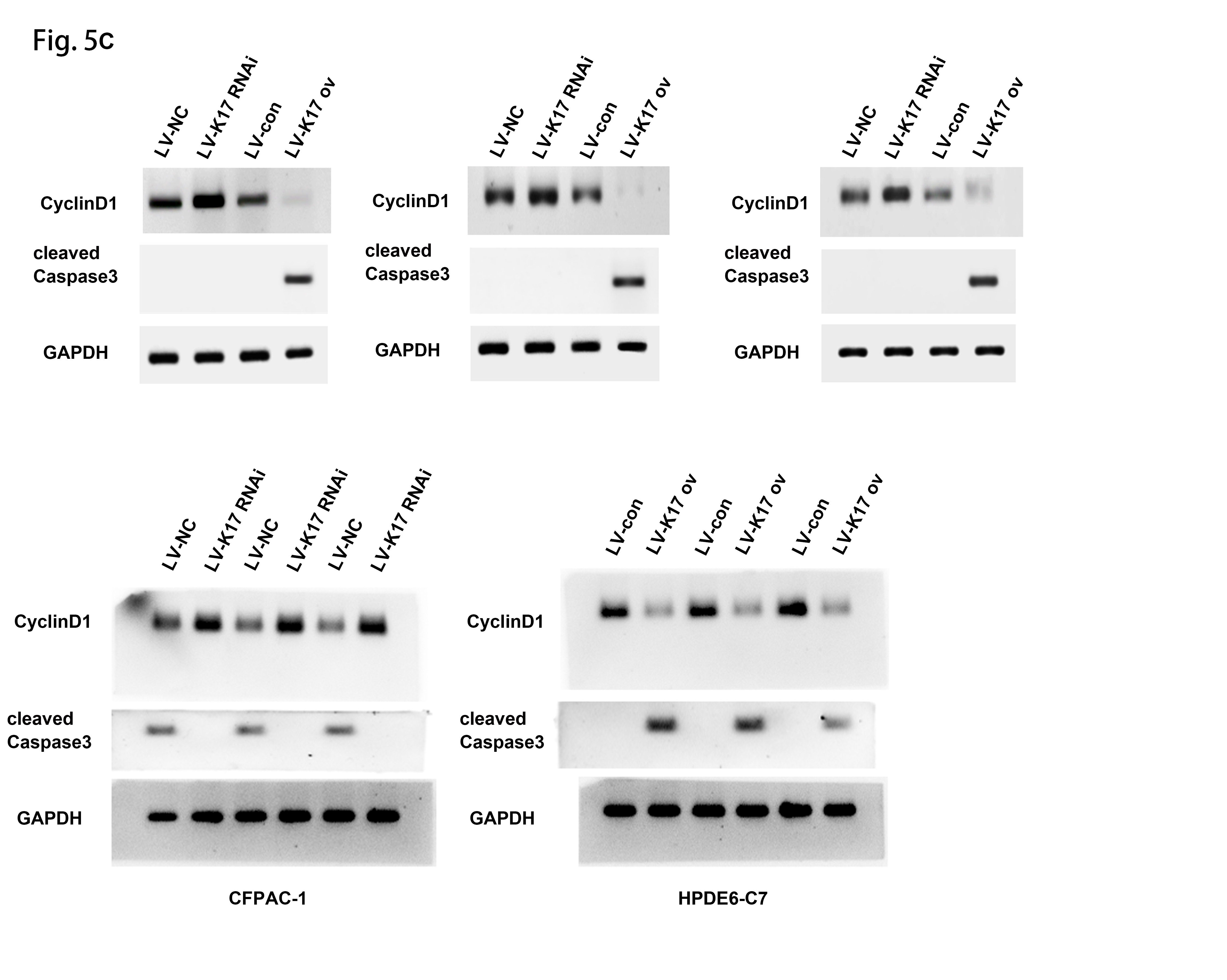

Supplement: Supplementary file 3 [file Image_3.JPEG]
